# The association between dietary fats and the incidence risk of cardiovascular outcomes: Tehran Lipid and Glucose Study

**DOI:** 10.1186/s12986-021-00624-6

**Published:** 2021-10-30

**Authors:** Zahra Gaeini, Parvin Mirmiran, Zahra Bahadoran, Maryam Aghayan, Fereidoun Azizi

**Affiliations:** 1 Nutrition and Endocrine Research Center, Research Institute for Endocrine Sciences, Shahid Beheshti University of Medical Sciences, No. 24, Shahid-Erabi St., Yeman St., Velenjak, Tehran, Iran; 2 Department of Clinical Nutrition and Dietetics, Faculty of Nutrition and Food Technology, National Nutrition and Food Technology Research Institute, Shahid Beheshti University of Medical Sciences, No. 24, Shahid-Erabi St., Yeman St., Velenjak, Tehran, Iran; 3 Endocrine Research Center, Research Institute for Endocrine Sciences, Shahid Beheshti University of Medical Sciences, Tehran, Iran

**Keywords:** Dietary fat, Saturated fatty acids, Mono-unsaturated fatty acids, Poly-unsaturated fatty acids, Cardiovascular disease

## Abstract

**Background:**

The association between dietary fats and the risk of cardiovascular disease (CVD) is under debate. We aimed to determine the potential effects of dietary saturated fats (SFA), mono-unsaturated (MUFA) and poly-unsaturated (PUFA) fatty acids on the occurrence of CVD in an adult Asian population.

**Methods:**

This study was conducted within the framework of the Tehran Lipid and Glucose Study on 2809 CVD-free adults, aged ≥ 19 years. Dietary intakes of fats were estimated using a validated 168-items semi-quantitative food frequency questionnaire, at baseline. Adjusted hazard ratios and 95% confidence intervals of CVD were calculated in tertile categories of dietary fats. The risk of CVD was estimated with multivariable Cox regression for the substitution of total fat or SFA with other macronutrients.

**Results:**

During 10.6 years of follow up, the incidence rate of CVD events was 7.1%. Mean (± SD) age of the participants was 39 (± 14) years and 43.9% were men. Total fat, animal and plant sources of fats were not associated with risk of CVD events. No significant associations were found between total SFA, lauric acid, myristic acid, stearic acid, palmitic acid as well as MUFA and PUFA and CVD incidence. Substitutions of total fats or SFA with other macronutrients were not associated with CVD risk.

**Conclusions:**

In this study, no significant associations were found between dietary fats and CVD risk. Considering the emerging body of literature that suggests no association between fats and CVD risk, reconsideration of dietary recommendations regarding low fat diets to prevent CVD, seems to be essential.

**Supplementary Information:**

The online version contains supplementary material available at 10.1186/s12986-021-00624-6.

## Introduction

Traditional dietary guidelines generally recommended a low-fat diet (less than 30% of energy) and limiting saturated fatty acid (SFA) intake (less than 10% of energy) to prevent cardio-vascular disease (CVD) [1]. The recommendations on lowering fats are largely based on the hypothesis which suggested a linear association between dietary fatty acids and serum low density lipoprotein (LDL)-cholesterol, and the association between LDL-C concentration and development of atherosclerosis and cardiovascular events [2].

Despite guidelines to restrict total fat and SFA intake, findings from many prospective cohort studies have not supported any significant association between dietary fats and CVD. A large prospective cohort study in 18 countries reported that total fat and SFA intakes were not significantly associated with CVD [3]. A recent meta-analysis that included 43 cohort studies also showed no significant association between total fat, SFA, mono-unsaturated (MUFA) and poly-unsaturated (PUFA) fatty acids with the risk of CVD events [4]. Also, few studies investigated the association between individual saturated fatty acids (such as lauric acid, myristic acid, palmitic acid and stearic acid) in diet and CVD incidence, they had inconsistent results [5–7]. Prospective studies regarding the substituting macronutrients generally showed conflicting results, some of them reported a beneficial effect on CVD risk when SFA were replaced by PUFAs [8–10], whereas replacement of SFA with carbohydrates, mostly low quality carbohydrates, had clear adverse effect on cardio-metabolic risk factors [9, 11]. Obviously, reconsideration of dietary guidelines in case of the quantity and quality of fats and replacement them with other macronutrients, seems to be essential.

Here we aimed to evaluate the potential association between dietary total, saturated and unsaturated fats, individual fatty acids, as well as animal and plant sources of fats, with risk of CVD events. Also, we investigated the association between fats and CVD, taking into account substitution with other macronutrients.

## Methods

### Study population

**Fig. 1:**
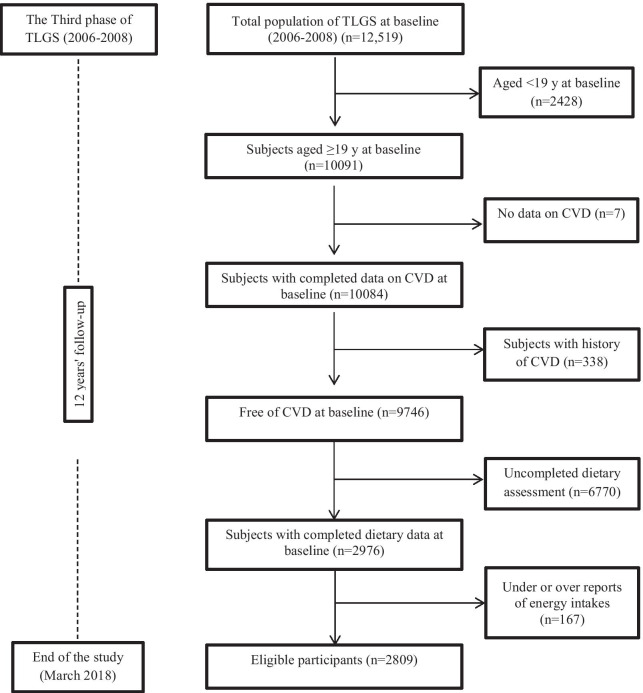
The diagram of the study

For the present analysis we used data collected from the Tehran Lipid and Glucose Study (TLGS). Participants of TLGS, who are a sample of residents from district 13, Tehran, Iran, have been followed up since 1999, and data recollection is designed to be in 3-year intervals [12]. The third phase of TLGS (2006–2008) was conducted with participation of 12,519 adult from both genders, of which 4920 adult were randomly selected to complete the dietary assessment. The randomization was based on the participant’s age and sex, to alleviate the cost and complexity dietary data collection in a large population. Finally, dietary data were available for 3462 adult men and women who agreed to participate and completed the food frequency questionnaire (FFQ). Its notable that the characteristics of participants who completed the FFQ were similar to those of the total population in the third phase of TLGS [13]. After exclusion of the participants who aged < 19 years, as well as participants with history of CVD outcomes, subjects who had incomplete data in terms of CVD, demographics, anthropometrics and biochemical measurements, 2976 healthy adults remained. From those, participants with under- or over-reports of energy intakes (< 800 kcal/d or > 4200 kcal/d, respectively), or with specific diets were excluded. Final study population was 2809 adults (Fig. 1); the remaining eligible participants were followed up to the end of the study (March 2018). Median period of follow-up was 10.6 years from the baseline examination.

### Anthropometric and demographic measures

Anthropometric measurements; including weight, height and waist circumference of participants, as well as demographic variables were collected by trained interviewers of TLGS, detailed information have been described elsewhere [14]. Systolic (SBP) and diastolic (DBP) blood pressure measurements were conducted using a standard mercury sphygmomanometer calibrated by the Iranian Institute of Standards and Industrial Researches [15], details regarding blood pressure measurements have been described elsewhere [16].

Modifiable Activity Questionnaire (MAQ) was used to evaluate levels of physical activity of participants; the frequency and time spent on light, moderate, hard and very hard intensity activities according to the list of common activities of daily life over the past year were documented. Physical activity levels expressed as metabolic equivalent hours per week (MET-min/week) [17]. Reliability and validity of the Persian version of the MAQ have previously been investigated [18]. Scores ≤ 600 METs-min/week were considered as having low physical activity, and scores > 600 METs-min/week were considered as moderate and high physical activity.

### Biochemical measures

Participants' blood samples were taken after 12–14 h of overnight fasting, at 7:00 and 9:00 AM. Details on biochemical measurements (including fasting plasma glucose (FPG), 2-h post-challenging plasma glucose (2 h-PCPG), serum triglyceride (TG), total cholesterol and High-Density Lipo-protein Cholesterol (HDL-C)) have been described elsewhere [14].

### Dietary assessment

Usual dietary intakes were assessed by a validated 168-item food frequency questionnaire (FFQ). Participants were asked to designate their intake frequency for each food item consumed during the past year on a daily, weekly, or monthly basis. The frequencies were then converted to daily intakes, and portion sizes reported in household measures converted to grams [13]. The USDA food composition table was used to obtain the amount of energy per grams of each type of food, and the amount of fat compositions. Validity of the FFQ has been previously evaluated [19].

### Definition of terms and outcomes

Details of the data collection for CVD outcomes have been described elsewhere [20]. Participants who had at least one of the coronary heart disease (CHD) event, stroke (a new neurological deficit that lasted ≥ 24 h), or CVD deaths (definite fatal myocardial infraction (MI), definite fatal CHD, and definite fatal stroke) were considered as a patient with CVD outcome [21]. CHD events included cases of definite MI (diagnostic ECG and biomarkers), probable MI (positive ECG findings plus cardiac symptoms or signs plus missing biomarkers or positive ECG findings plus equivocal biomarkers), and angiographic proven CHD. Also, subjects with history of CVD were considered as any subject who had a previous ischemic heart disease and/or cerebro-vascular accident. Hypertension (HTN) was defined as SBP ≥ 140 mmHg, or DBP ≥ 90 mmHg, or self-report of taking blood pressure lowering medications [22]. Type 2 diabetes patients were defined as participants who met at least one of these criteria: FPG ≥ 126 mg/dL, 2 h-PCPG ≥ 200 mg/dL, or self-reported of taking anti-diabetic medications [23].

### Statistical analyses

Mean (± SD) values and frequencies (%) of baseline characteristics of participants were compared according to incidence of CVD outcome, using t-test for normally distributed continuous variables, the Chi squared test for categorical variables, and the Mann–Whitney test for skewed variables. To handle missing values, the multiple imputations by chained equations (MICE) method was employed. The incidence of CVD over the follow-up period was considered as a dichotomous variable (yes/no) in the models. Dietary intakes of total fat, animal and plant sources of fats, total SFA, lauric acid, myristic acid, palmitic acid, stearic acid, total MUFA and PUFA, total omega-3 and omega-6 and their ratio were categorized into tertiles, and the first tertile was considered as reference.

Cox proportional hazards regression models with person-years as the underlying time metric were used to estimate HRs and 95% CIs for the associations between intakes of different types of fats and CVD incidence. Time to event for CVD was defined as time to end of follow-up (censored cases) or time to having an event, whichever occurred first. We censored participants at the time of death due to non-CVD causes, at time of leaving the district, or end of study follow-up (March 2018). For the censored and lost to follow-up subjects, the survival time was the interval between the first and the last observation dates. The proportional hazard assumption of the multivariable Cox model was assessed using Schoenfelds global test of residuals.

To obtain the final multivariable models and determine confounding variables, we performed a univariate analysis. Variables with P_E_ less than 0.2 in the univariate analyses were selected as confounders. Potential confounders, adjusted in the Cox models, include sex (men/women), waist circumference (cm), body mass index (BMI) (kg/m^2^), HTN (yes/no), CVD risk score (continuous) [35], physical activity level (low/high), total energy (kcal/d), total fat and fiber intakes (g/d), dietary cholesterol intake (mg/d), and daily intake of tea and coffee (ml/d). The CVD risk score calculated based on age, total cholesterol, HDL-C, SBP, treatment for HTN, smoking, and type 2 diabetes status, which has been validated among Iranian population [36]. Adjustment of CVD risk score, as a continuous potential risk factor of CVD events, improved the stability of our models due to the limited number of events during the study follow-up. To evaluate the dose–response associations between dietary fats and CVD risk, the method of restricted cubic spline was used [24].

The association of substituting 5% of energy from total fat for 5% of energy from carbohydrate and protein, or 1% of energy from saturated fat for 1% of energy from other individual fats or macronutrients, with risk of CVD event was assessed by including them as continuous variables in the same multivariate model, which also contained non-dietary covariates and total energy intake. The difference in their coefficients and in their own variances and covariance were used to estimate the substitution associations [25].

All statistical analyses were performed using the Statistical Package for Social Science (version 20; IBM Corp., Armonk, NY, USA) and STATA version 16 SE (STATA Inc., TX, USA), *P*-values < 0.05 being considered significant.

## Results

Mean age (± SD) of participants was 39.56 ± 14.02 years, and 43.9% of participants were men. Median (inter-quartile range) of follow-up duration was 10.6 (9.9–11.1) years, the incidence rate of CVD events during that time was 7.1%. Mean (± SD) dietary intakes of total fat, total SFA, lauric acid, myristic acid, palmitic acid, stearic acid, MUFA, PUFA, omega-3 and omega-6 were 31.32 (± 7.07), 10.54 (± 4.70), 0.35 (± 0.23), 0.87 (± 0.39), 4.90 (± 1.36), 2.00 (± 0.54), 10.87 (± 2.88), 6.55 (± 2.35), 0.52 (± 0.25), 5.66 (± 2.22), as percentages of total energy intake, respectively.

Baseline characteristics of participants are shown in Table 1. Compared with participants who had no CVD events, participants with CVD tended to be older, more likely to be men and current smokers, had higher BMI, waist circumference, TG/HDL ratio, serum concentration of TG, total cholesterol, HDL-C, LDL-C and higher incidence of HTN (*P* for all < 0.05). Also, participants who had CVD outcomes, had significantly lower daily intakes of total fat, total SFA, lauric acid, myristic acid, stearic acid, total MUFA, animal fats and total cholesterol and significantly higher daily intake of tea and coffee at baseline (*P* for all < 0.05). There was no significant difference in dietary intakes of total energy, palmitic acid, total PUFA, omega-3, omega-6, omega-6/omega-3 ratio, and plant fats between the two groups.

**Table 1:** Baseline characteristics of participants according to CVD events

Baseline characteristics	CVD^+^ (n = 198)	CVD^−^ (n = 2611)	P_value
Age, year	57.06 ± 10.25	38.22 ± 13.36	< 0.001
Male, %	65.7	42.3	< 0.001
Current smoker, %	16.2	8.8	0.003
Low physical activity*, %	40.2	38.2	0.591
Body mass index, kg/m^2^	28.48 ± 4.61	26.84 ± 4.88	< 0.001
Waist circumference, cm	97.91 ± 10.67	88.81 ± 13.27	< 0.001
Hypertension, %	78.9	21.1	< 0.001
TG to HDL ratio^†^	4.26 (2.98–6.37)	2.82 (1.79–4.53)	< 0.001
Serum TG (mg/dL)^†^	165 (123–211)	117 (81–170)	< 0.001
Serum cholesterol (mg/dL)	208.7 ± 42.14	184.4 ± 37.98	< 0.001
Serum LDL-C (mg/dL)	134.0 ± 36.25	113.7 ± 32.16	< 0.001
Serum HDL-C (mg/dL)	39.48 ± 8.59	43.14 ± 10.40	< 0.001
Total energy intake, kcal/day	2240 ± 726	2257 ± 719	0.757
Total fat intake, g/day^†^	66.59 (51.90–88.95)	73.77 (56.67–96.53)	0.009
Total protein intake, g/day^†^	72.40 (59.44–88.77)	72.86 (57.66–93.05)	0.659
Total carbohydrate intake, g/day^†^	314 (253–395)	308 (241–388)	0.315
Total saturated fats intake, g/day^†^	21.10 (16.99–27.97)	24.23 (18.40–32.44)	< 0.001
Lauric acid (12:0), g/day^†^	0.63 (0.43–0.87)	0.74 (0.50–1.06)	< 0.001
Myristic acid (14:0), g/day^†^	1.75 (1.14–2.41)	1.97 (1.37–2.71)	0.001
Palmitic acid (16:0), g/day^†^	10.44 (8.01–14.14)	11.37 (8.59–14.84)	0.060
Stearic acid (18:0), g/day^†^	4.15 (3.14–5.40)	4.65 (3.48–6.23)	< 0.001
Total mono-unsaturated fats intake, g/day^†^	22.48 (17.62–30.42)	25.34 (19.19–33.42)	0.003
Total poly-unsaturated fats intake, g/day^†^	14.10 (10.30–19.65)	15.22 (10.76–20.03)	0.103
Total omega-3, g/day^†^	1.14 (0.74–1.56)	1.18 (0.81–1.60)	0.320
Total omega-6, g/day^†^	11.92 (8.53–17.05)	13.10 (9.08–17.41)	0.080
Omega-6/ omega-3 ratio^†^	11.11 (9.05–13.51)	11.39 (9.35–13.41)	0.370
Animal fats, g/day^†^	33.43 (26.03–45.33)	38.78 (28.77–52.53)	< 0.001
Plant-base fats, g/day^†^	32.51 (20.73–43.86)	33.73 (21.90–45.45)	0.285
Total cholesterol intake, mg/day^†^	172 (135–242)	197 (142–273)	0.005
Tea and coffee intake, mL/day^†^	750 (251–1000)	501 (251–758)	0.024

Values are shown as mean ± SD and number (%), for continuous and categorical variables, respectively

*TG* Triglyceride, *LDL-C* low density lipoprotein cholesterol, *HDL-C* high density lipoprotein cholesterol

^†^Values are shown as median (Interquartile range)

^*^Modifiable activity questionnaire (MAQ) scores ≤ 600 METs-min/week was considered as low physical activity

The HRs (95% CI) of CVD events across categories of dietary fat intake are shown in Table 2. After adjustment for confounding variables, there was no significant association between total fat, animal and plant sources of fats with the risk of CVD (HR 0.80, 95% CI 0.46–1.37; *P* for trend = 0.396 for total fat, HR 0.80, 95% CI = 0.49–1.28; *P* for trend = 0.459 for animal sources of fats, HR 0.87, 95% CI 0.56–1.35; *P* for trend = 0.583 for plant sources of fats).

**Table 2:** Risk of CVD events according to dietary fat sources

Food group	Tertile 1	Tertile 2	Tertile 3	P for trend
*Total fat*				
Median intake (g/day)	49.95	75.12	110.39	
Range (g/day)	14.64–62.75	62.80–90.25	90.29–246.9	
HR (95% CI) Crude model	1.00	0.69 (0.49–0.98)	0.65 (0.46–0.93)	0.016
HR (95% CI) Model 1	1.00	0.73 (0.51–1.04)	0.85 (0.60–1.21)	0.313
HR (95% CI) Model 2	1.00	0.68 (0.46–1.01)	0.80 (0.46–1.37)	0.396
*Animal fats*				
Median intake (g/day)	17.79	29.86	48.23	
Range (g/day)	0–24.44	24.44–36.88	36.89–164.88	
HR (95% CI) Crude model	1.00	0.75 (0.53–1.06)	0.54 (0.37–0.79)	0.001
HR (95% CI) Model 1	1.00	0.96 (0.68–1.34)	0.78 (0.53–1.13)	0.205
HR (95% CI) Model 2	1.00	0.95 (0.66–1.38)	0.80 (0.49–1.28)	0.459
*Plant fats*				
Median intake (g/day)	17.64	33.66	51.94	
Range (g/day)	4.34–25.84	25.85–41.10	41.14–180.91	
HR (95% CI) Crude model	1.00	0.71 (0.50–1.02)	0.77 (0.54–1.10)	0.140
HR (95% CI) Model 1	1.00	0.78 (0.54–1.11)	0.94 (0.66–1.34)	0.696
HR (95% CI) Model 2	1.00	0.77 (0.53–1.11)	0.87 (0.56–1.35)	0.583

Data are hazard ratio (95% CI); proportional hazard Cox regression was used. HR, Hazard Ratio; CI, Confidence Interval; CVD, Cardio-vascular Disease

Model 1 was adjusted for sex, waist circumference, body mass index, CVD score, hypertension and physical activity. Model 2 was additionally adjusted for total energy (kcal/d), fiber (g/d), cholesterol (mg/d), tea and coffee intake (ml/d)

The risk of CVD across tertile categories of dietary SFA are shown in Table 3. In the crude models, higher intakes of total SFA, as well as lauric acid, myristic acid and stearic acid were associated with lower risk of CVD events (*P* for all < 0.05). However, there were no significant associations between total SFA or individual saturated fatty acids and CVD risk in the fully adjusted models (HR 0.75, 95% CI 0.45–1.23; *P* for trend = 0.263 for total SFA, HR 0.79, 95% CI 0.52–1.18; *P* for trend = 0.377 for lauric acid, HR 0.94, 95% CI 0.63–1.40; *P* for trend = 0.874 for myristic acid, HR 1.05, 95% CI 0.71–1.54; *P* for trend = 0.800 for palmitic acid, HR 0.70, 95% CI = 0.42–1.16; *P* for trend = 0.150 for stearic acid). The HRs (with 95% CI) of CVD events across tertile categories of dietary unsaturated fats are shown in Table 4. Although higher intake of MUFA was associated with lower risk of CVD events in the crude model (HR 0.64, 95% CI 0.45–0.90; *P* for trend = 0.009), there was no significant association between total MUFA or PUFA, as well as omega-3, omega-6 fats and their ratio, and CVD risk, after adjustment for potential confounders (HR 0.90, 95% CI 0.53–1.55; *P* for trend = 0.667 for total MUFA, HR 1.08, 95% CI 0.67–1.73; *P* for trend = 0.680 for total PUFA, HR 1.52, 95% CI 0.98–2.33; *P* for trend = 0.057 for omega3, HR 1.02, 95% CI 0.64–1.63; *P* for trend = 0.893 for omega6, HR 0.91, 95% CI 0.64–1.31; *P* for trend = 0.626 for omega6/omega3 ratio).

**Table 3:** Risk of CVD events according to dietary intakes of saturated fats

Saturated fats	Tertile 1	Tertile 2	Tertile 3	P for trend
*Total saturated fats*				
Median intake (% of energy)	7.74	10.17	13.12	
Range (% of energy)	2.30–9.04	9.05–11.38	11.38–196.91	
CVD event (n)	93	62	43	
HR (95% CI) Crude Model	1.00	0.74 (0.53–1.03)	0.45 (0.30–0.66)	< 0.001
HR (95% CI) Model 1	1.00	0.89 (0.64–1.24)	0.68 (0.46–1.01)	0.061
HR (95% CI) Model 2	1.00	0.92 (0.63–1.34)	0.75 (0.45–1.23)	0.263
*Lauric acid*				
Median intake (% of energy)	0.19	0.31	0.47	
Range (% of energy)	0.02–0.25	0.25–0.37	0.37–4.53	
CVD event (n)	87	70	41	
HR (95% CI) Crude Model	1.00	0.94 (0.68–1.32)	0.54 (0.36–0.80)	< 0.001
HR (95% CI) Model 1	1.00	1.27 (0.90–1.78)	0.75 (0.51–1.11)	0.228
HR (95% CI) Model 2	1.00	1.31 (0.93–1.84)	0.79 (0.52–1.18)	0.377
*Myristic acid*				
Median intake (% of energy)	0.52	0.83	1.19	
Range (% of energy)	0.06–0.68	0.68–0.97	0.97–4.81	
CVD event (n)	85	71	42	
HR (95% CI) Crude Model	1.00	0.85 (0.61–1.20)	0.65 (0.45–0.94)	0.007
HR (95% CI) Model 1	1.00	1.09 (0.77–1.54)	0.85 (0.59–1.23)	0.452
HR (95% CI) Model 2	1.00	1.17 (0.83–1.67)	0.94 (0.63–1.40)	0.874
*Palmitic acid*				
Median intake (% of energy)	3.73	4.74	6.03	
Range (% of energy)	1.87–4.27	4.27–5.28	5.28–14.42	
CVD event (n)	77	68	53	
HR (95% CI) Crude Model	1.00	0.60 (0.42–0.87)	0.77 (0.55–1.09)	0.113
HR (95% CI) Model 1	1.00	0.81 (0.56–1.17)	0.96 (0.68–1.36)	0.786
HR (95% CI) Model 2	1.00	0.85 (0.57–1.26)	1.05 (0.71–1.54)	0.800
*Stearic acid*				
Median intake (% of energy)	1.51	1.96	2.49	
Range (% of energy)	0.67–1.74	1.74–2.19	2.19–6.69	
CVD event (n)	90	65	43	
HR (95% CI) Crude Model	1.00	0.61 (0.44–0.85)	0.45 (0.31–0.65)	< 0.001
HR (95% CI) Model 1	1.00	0.80 (0.57–1.12)	0.69 (0.48–1.01)	0.052
HR (95% CI) Model 2	1.00	0.79 (0.54–1.16)	0.70 (0.42–1.16)	0.150

Data are hazard ratio (95% CI); proportional hazard Cox regression was used. HR, Hazard Ratio; CI, Confidence Interval; CVD, Cardio-vascular Disease

Model 1 was adjusted for sex, waist circumference, body mass index, CVD score, hypertension and physical activity. Model 2 was additionally adjusted for total energy intake (kcal/d), total fat (g/d), total fiber (g/d), cholesterol (mg/d), tea and coffee intake (ml/d)

**Table 4:** Risk of CVD events according to dietary intakes of unsaturated fats

Unsaturated fats	Tertile 1	Tertile 2		Tertile 3	P for trend
*Mono-unsaturated fats*					
Median intake (% of energy)	8.27	10.62	13.48		
Range (% of energy)	3.13–9.54	9.55–11.79	11.80–22.83		
CVD event (n)	91	62	45		
HR (95% CI) Crude Model	1.00	0.68 (0.49–0.96)	0.64 (0.45–0.90)		0.009
HR (95% CI) Model 1	1.00	0.84 (0.60–1.20)	0.80 (0.56–1.15)		0.209
HR (95% CI) Model 2	1.00	0.89 (0.60–1.31)	0.90 (0.53–1.55)		0.667
*Poly-unsaturated fats*					
Median intake (% of energy)	4.42	6.16	8.54		
Range (% of energy)	1.99–5.34	5.34–7.12	7.12–19.73		
CVD event (n)	77	68	53		
HR (95% CI) Crude Model	1.00	1.04 (0.74–1.46)	0.87 (0.61–1.24)		0.455
HR (95% CI) Model 1	1.00	1.29 (0.91–1.82)	0.99 (0.68–1.43)		0.986
HR (95% CI) Model 2	1.00	1.32 (0.91–1.93)	1.08 (0.67–1.73)		0.680
*Total Omega 3*					
Median intake (% of energy)	0.32	0.49	0.68		
Range (% of energy)	0.05–0.41	0.41–0.57	0.57–3.78		
CVD event (n)	72	55	71		
HR (95% CI) Crude Model	1.00	0.77 (0.53–1.10)	1.02 (0.73–1.43)		0.897
HR (95% CI) Model 1	1.00	0.80 (0.55–1.16)	1.14 (0.81–1.61)		0.449
HR (95% CI) Model 2	1.00	0.92 (0.62–1.38)	1.52 (0.98–2.33)		0.057
*Total Omega 6*					
Median intake (% of energy)	3.65	5.30	7.54		
Range (% of energy)	1.59–4.53	4.53–6.21	6.22–17.88		
CVD event (n)	78	68	52		
HR (95% CI) Crude Model	1.00	0.96 (0.68–1.36)	0.89 (0.62–1.26)		0.507
HR (95% CI) Model 1	1.00	1.11 (0.78–1.57)	0.97 (0.67–1.40)		0.888
HR (95% CI) Model 2	1.00	1.11 (0.76–1.61)	1.02 (0.64–1.63)		0.893
*Omega 6/omega 3 ratio*					
Median intake (% of energy)	8.47	11.36	14.43		
Range (% of energy)	1.59–10.07	10.07–12.67	12.68–58		
CVD event (n)	73	59	66		
HR (95% CI) Crude Model	1.00	0.77 (0.54–1.10)	0.90 (0.64–1.26)		0.513
HR (95% CI) Model 1	1.00	0.90 (0.63–1.30)	0.99 (0.69–1.40)		0.926
HR (95% CI) Model 2	1.00	0.85 (0.59–1.24)	0.91 (0.64–1.31)		0.626

Data are hazard ratio (95% CI); proportional hazard Cox regression was used. HR, Hazard Ratio; CI, Confidence Interval; CVD, Cardio-vascular Disease

Model 1 was adjusted for sex, waist circumference, body mass index, CVD score, hypertension and physical activity. Model 2 was additionally adjusted for total energy intake (kcal/d), total fat (g/d), total fiber (g/d), cholesterol (mg/d), tea and coffee intake (ml/d)

Additionally, the adjusted HR (with 95% CI) of CVD according to continuous form of fats intake were 0.96 (0.88–1.05) for total fat (per 10 g/d), 0.93 (0.83–1.05) for total SFA (per 5 g/d), 0.91 (0.81–1.02) for MUFA (per 5 g/d), 0.94 (0.82–1.07) for PUFA (per 5 g/d). Also, a restricted cubic spline was conducted to evaluate the dose–response associations of dietary fats and risk of CVD events. There was no significant non-linear association between dietary intake of total fat, animal and plant-based fats, SFA, MUFA, PUFA and risk of CVD (Additional file 1: Fig. 1).

**Fig. 2:**
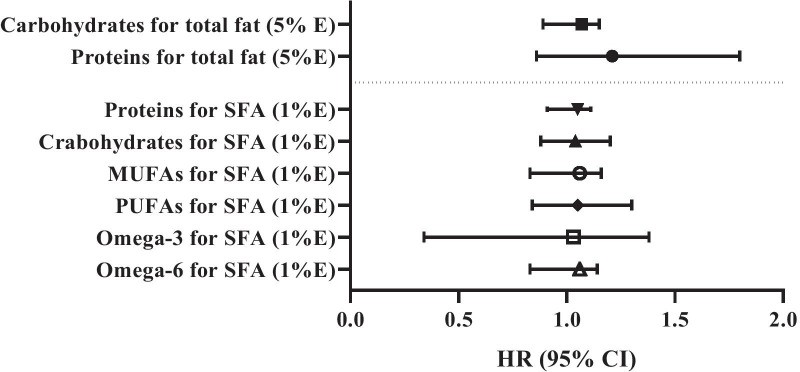
Substitution of 5% energy from total fat for 5% energy from proteins and carbohydrates, substituting 1% energy from saturated fat for 1% energy from carbohydrate, protein, PUFA, MUFA, omega-3 and omega-6 while keeping total energy intake constant, and risk of CVD events

Substitution analysis for estimating the replacement of 5% of energy from total fat with 5% of energy from carbohydrates or proteins, and substituting 1% of energy from SFA for 1% of energy from carbohydrate, protein, PUFA, MUFA, omega-3 and omega-6 while keeping total energy intake constant, and risk of CVD events are shown in Fig. 2. Substituting total fat or SFAs for other macronutrients or other fats were not in relationship with risk of CVD in the fully adjusted model.

## Discussion

In this prospective cohort study, no significant associations were found for total fat, animal or plant sources of fats, and CVD events risk. Also, usual intake of total SFA, MUFA, PUFA, omega-3 and omega-6 intakes were not associated with CVD risk. SFAs with different carbon length also had no association with CVD risk. The iso-caloric substitutions of total fat or SFA with other macronutrients were not associated with CVD risk.

The non-significant inverse association observed between dietary total fat and CVD risk is in line with the results from two studies [3, 26], which suggested no significant association. A meta-analysis of 45 studies showed that higher intakes of total fat, SFA, MUFA or PUFA were not associated with risk of CVD events or mortality, pooled HRs estimates (95% CIs) for CVD incidence and mortality in highest *vs.* lowest levels of dietary fats were 0.97 (0.93–1.01) for total fat, 0.97 (0.93–1.02) for total SFA, 0.97 (0.93–1.01) for MUFA and 0.97 (0.93–1.004) for PUFA [4]. Dose response analysis of this study suggested a non-significant linear relationship between total fat intake and CVD risk (HR 0.99, 95% CI 0.97–1.01) for an increment of 5% of energy from total fat) [4]. Median intake of total fat in the present study was 30.92% from total energy, while the previous related studies reported varied amounts for median intake of total fat, from 23.44% [3] to 38.42% [26] and 39.3% [27] from total energy in another study, it seems that the discrepancies observed between the study’s results may be related to the different dietary habits of the participants. Though the popular accepted views of dietary advices to avoid fats intake are continue to evolve, prospective cohort studies with long period of time (approximately 4 to 20 years) and meta-analysis of clinical trials did not detect a positive association between dietary total fat and CVD risk. Considering the results of the recent studies, it seems that it is necessary to reconsider the dietary guidelines recommendations to emphasize more on the quality of macronutrients, instead of focusing on low fat diets.

Our findings for the association between total SFA and CVD events are in line with the results from a meta-analysis of 21 prospective studies, which reported a non-significant inverse association between SFA and CVD incidence [28]. However, our results are conflict with a recent meta-analysis of 15 randomized controlled trials that found a positive association between SFA intake and CVD events [29]. The inconsistencies in the results may be explained by the differences in study designs; observational or experimental designs. Another explanation for our findings may be that the main sources of SFA in present study were milk and milk products, which had cardio-protective effects in some studies [30, 31]. On the other hand, different amount of daily SFA intake by participants (10.17% from total energy in our study vs. 14.9% from total energy in another related studies [32]) may explain the inconsistent findings.

When we differentiated SFAs according to chain lengths of fatty acids, we observed that higher intakes of lauric, myrisic, palmitic and stearic acid had no significant effect on CVD incidence risk. In contrast to our findings, a study [7] showed a significant positive association between dietary intakes of myristic and stearic acid and risk of CHD. In another prospective study, higher intake of palmitic acid was associated with higher risk of CHD, and there were no associations for SFAs with other chain lengths [33], the results are partly in line with our findings. A large body of evidence showed that palmitic acid-rich fats could increase total cholesterol and LDL-C concentration, and palmitic acid is a well-known risk factor for CHD [34].

Although in our study we observed no significant association between dietary MUFA or PUFA and CVD risk, the association in previous studies was conflicting and not conclusive. In line with our findings, a large cohort study in 18 countries with a median follow-up of 7.4 years suggested no significant association between MUFA and PUFA intake and CVD risk [3]. In a recent meta-analysis of 45 cohort studies, dietary MUFA and PUFA intakes were not significantly associated with CVD risk; however, sub-group analysis demonstrated that in studies with follow-up duration more than 10 years, the risk of CVD incidence was reduced by 5% in the highest compared with the lowest categories of PUFA intake [4]. These may indicate the need for cohort studies with longer follow up time to clarify the effect of PUFA or MUFA on CVD risk. Furthermore, while several clinical trials and cohort studies demonstrated cardio-protective effects of omega-3 PUFA supplements or fish consumption [35–38], no effect [39] or adverse effect [40, 41] have been found in other studies, as we found. On the other hand, the omega-6 to omega-3 ratio in human diet is a very important issue for health, because of the competition between them for desaturation enzymes activity and metabolism. Modern dietary patterns, such as Western dietary pattern, provide the highest omega-6 to omega-3 ratio, ranging from 8:1 to 20:1 or even higher, instead of the healthy recommended ratio of 1:1 [42]. The mean (± SD) of omega-6 to omega-3 ratio in our population was 11.81 (± 4.55).

We have not found any significant association between substitution of total fat or SFA with other fatty acids and macronutrients, on CVD risk. The results of previous studies regarding the effect of substitution dietary fats on CVD risk are conflicting and not conclusive. Similar to our findings, some previous studies suggested that replacing total SFA with carbohydrate [33], MUFA [8, 43] and PUFA [33] had no beneficial effects on CVD risk, while some others suggested a higher risk of CVD for replacing total SFA with total carbohydrate, MUFA, PUFA or animal protein [32, 44]. The inconsistent findings may be explained by the differences in fatty acid sources in different populations, or it may relate to the method used in the studies to investigate the association of substitutions. The method we used for substitution, as in the most of the observational studies, may not be ideal due to the fact that substitution of macronutrients was statistically modeled using dietary data that were derived at one time point and the replacements actually were a simultaneous comparison. Changes in dietary intakes during the follow-up period could affect the associations.

Altogether, considering the contradictions in studies regarding the association between dietary fats and CVD incidence, and given to the importance of early prevention strategies for reduce the risk of CVD events, reconsideration of dietary recommendations regarding low fat diets to prevent CVD, seems to be essential. The first Dietary Guideline for Americans in 1980 recommended reducing total and SFA intake, since that time the intake of total fat and SFA has steadily decreased as a percent of calories and it has largely reflected a corresponding increase in dietary carbohydrate [9]. These dietary trends not only did not reduce the incidence of CVD, but it also increased the incidence of CVD and other chronic diseases in recent decades. Along with recent advances in nutrition sciences, the 2015–2020 *Dietary Guidelines for Americans* has removed the limitation on total fat intake and placed emphasis on the types and quality of fats consumed within the context of a healthy dietary pattern [45].

Strengths of this study include the prospective design of the study, long follow-up period, validation of FFQ, representativeness of the general population and consideration of different fat sources. However, some limitations of the present study should be taken into account. First, despite adjustment for a wide range of potential confounders in our analysis, the residual confounders from unknown factors should be considered. Second, as any prospective study, changes in an individual’s diet and other CVD risk factors during the study follow-up, may lead to some degree of misclassification and biased estimated hazard ratios toward the null. Third, as with any observational study, we cannot report any causation between dietary fats and risk of incident CVD. Finally, potential measurement errors in estimated of food and nutrient intakes, due to the self-report questionnaire, could be as a limitation point of the study.

## Conclusions

To conclude, we did not observe any significant association between different types of dietary fats and risk of CVD incidence in our population. Further prospective and clinical trial studies are needed to clarify the association between dietary macronutrients and CVD risk, and to reconsider the dietary guidelines to prevent CVD events.

## Supplementary Information

**Additional file 1**. Association between dietary total fat, animal and plant-based fat, saturated and unsaturated fats and risk of CVD events based on restricted cubic spline model.

## Data Availability

The datasets used and/or analyzed during the current study available from the corresponding author on reasonable request.

## Abbreviations

BMI: Body Mass Index
CHD: Coronary Heart Disease
CI: Confidence Interval
CVD: Cardiovascular Disease
DBP: Diastolic Blood Pressure
FFQ: Food Frequency Questionnaire
HDL-C: High-Density Lipoprotein Cholesterol
HR: Hazard Ratio
HTN: Hypertension
LDL-C: Low-Density Lipoprotein Cholesterol
MET: Metabolic Equivalent
MUFA: Mono-Unsaturated Fatty Acid
PUFA: Poly-Unsaturated Fatty Acid
SFA: Saturated Fatty Acid
SBP: Systolic Blood Pressure
TLGS: Tehran Lipid and Glucose Study
TG: Triglyceride
