# Supplementary material for: The association between dietary fats and the incidence risk of cardiovascular outcomes: Tehran Lipid and Glucose Study
Source: Nutr Metab (Lond). 2021 Oct 30;18:96. doi: 10.1186/s12986-021-00624-6 (PMC8557498; doi:10.1186/s12986-021-00624-6)
Supplement: Supplementary file 1 — Additional file 1. Association between dietary total fat, animal and plant-based fat, saturated and unsaturated fats and risk of CVD events based on restricted cubic spline model. [file 12986_2021_624_MOESM1_ESM.docx]

**Supplemental Figure 1.** Association between dietary total fat, animal and plant-based fat, saturated and unsaturated fats and risk of CVD events based on restricted cubic spline model.
